# A New School Physiology

**Published:** 1880-09

**Authors:** 


A NEW SCHOOL PHYSIOLOGY.—By Richard Dunglison, A.M., M.D., author of "Practitioner's Reference Book," Etc., Etc. Illustrated with 117 engravings. Porter & Coats, Philadelphia. 1880.

This is a work adapted to the elementary studies in Physiology. In a brief survey of it, we are led to believe that it fully meets the requirements for which it is intended. It is gotten up in suitable and usual style for school books, and whether in the academy, the country school-room, or the halls of college will serve the medium of much useful information on physiological subjects.

